# Reduced cytotoxicity of insulin-immobilized CdS quantum dots using PEG as a spacer

**DOI:** 10.1186/1556-276X-6-528

**Published:** 2011-09-23

**Authors:** KM Kamruzzaman Selim, Zhi-Cai Xing, Moon-Jeong Choi, Yongmin Chang, Haiqing Guo, Inn-Kyu Kang

**Affiliations:** 1 Department of Polymer Science and Engineering, Kyungpook National University, Daegu 702-701, South Korea; 2 Medical and Biological Engineering, Kyungpook National University, Daegu 702-701, South Korea; 3 Department of Diagnostic Radiology, Kyungpook National University, Dongin-dong, Daegu 700-422, South Korea; 4 College of Chemistry and Molecular Engineering, Peking University, Beijing 100871, China

**Keywords:** nanoparticles, immobilization, polyethylene glycol, insulin, cytotoxicity

## Abstract

Cytotoxicity is a severe problem for cadmium sulfide nanoparticles (CSNPs) in biological systems. In this study, mercaptoacetic acid-coated CSNPs, typical semiconductor Q-dots, were synthesized in aqueous medium by the arrested precipitation method. Then, amino-terminated polyethylene glycol (PEG) was conjugated to the surface of CSNPs (PCSNPs) in order to introduce amino groups to the surface. Finally, insulin was immobilized on the surface of PCSNPs (ICSNPs) to reduce cytotoxicity as well as to enhance cell compatibility. The presence of insulin on the surface of ICSNPs was confirmed by observing infrared absorptions of amide I and II. The mean diameter of ICSNPs as determined by dynamic light scattering was about 38 nm. Human fibroblasts were cultured in the absence and presence of cadmium sulfide nanoparticles to evaluate cytotoxicity and cell compatibility. The results showed that the cytotoxicity of insulin-immobilized cadmium sulfide nanoparticles was significantly suppressed by usage of PEG as a spacer. In addition, cell proliferation was highly facilitated by the addition of ICSNPs. The ICSNPs used in this study will be potentials to be used in bio-imaging applications.

## Introduction

Recently, quantum dots [CdS, CdSe, ZnS, CdTe, etc.] (Q-dots) have attracted tremendous interest as luminescent probes in biological and medical researches due to their unique optical and chemical properties [1]. Compared with traditional dyes and fluorescent proteins used as imaging probes, Q-dots have several advantages, such as tunable emission from visible to infrared wavelengths, broader excitation spectra, high quantum yield of fluorescence, strong brightness, photostability, and high resistance to photobleaching [2,3]. However, the potential applications of Q-dots in biology and medicine have been limited due to their cytotoxic effects [4]. Q-dots contain toxic components such as cadmium (from cadmium chalcogenide-based Q-dots) or lead (from lead chalcogenide-based Q-dots). Cd^2+ ^and Pb^2+ ^can be released from Q-dots, which would kill the cells [5]. Therefore, to enhance stability, the surface modification of Q-dots is required. For example, biomedical applications require high-quality water soluble and non-toxic Q-dots. So far, numerous surface modifications of Q-dots have been explored, including the attachment of mercaptoacetic acid [6], mercaptopropionic acid [7], mercaptobenzoic acid [8], and biocompatible and chemically functionalizable inorganic shells, such as silica or zinc sulfide [9]. All of these coatings can ensure the water solubility of Q-dots, but they are unable to enhance biocompatibility. Therefore, further coating with suitable water-soluble organic ligand/biomolecules is necessary to enhance the biocompatibility of Q-dots. To this end, Q-dots have been covalently linked with biorecognition molecules such as biotin [10], folic acid [11], peptides [12], bovine serum albumin [13], transferrin [14], antibodies [15], and DNA [16].

Polyethylene glycol (PEG) and its derivatives have been widely used as biomedical materials, such as drug delivery matrices and scaffolds for tissue engineering, due to their hydrophilicity, high solubility in aqueous and organic solvents, excellent biocompatibility, lack of toxicity and immunogenicity, and ease of excretion from living organisms. Among PEG derivatives, the most important one is amino-terminated PEG [17]. On the other hand, insulin, which reduces blood glucose levels, is often used for treating diabetic patients. However, insulin also acts as a growth factor, inducing cell proliferation [18,19]. It has been previously shown by research groups [18-21] that immobilized insulin stimulates cell growth more actively than free insulin. Therefore, introduction of PEG-insulin conjugate onto the surface of Q-dots through chemical bonding may confer the combined advantage of PEG and insulin. Introduction of PEG onto the surface of nanoparticles protects against unwanted agglomeration, makes them more biocompatible, and decreases their nonspecific intracellular uptake. On the contrary, insulin grafted onto the distal end of the PEG chain can enhance cells growth.

In this study, mercaptoacetic acid-coated cadmium sulfide nanoparticles (CSNPs), typical semiconductor Q-dots, were synthesized in aqueous medium by the arrested precipitation method at room temperature. Then, PEG with amino groups at both ends was reacted with carboxyl groups of CSNPs (PCSNPs) in order to introduce amino groups to the surface as well as to enhance biocompatibility. Finally, insulin was immobilized on the surface of PCSNPs (ICSNPs) to promote cell growth and further enhance biocompatibility. The surface properties of CSNPs and ICSNPs were characterized by X-ray diffraction (XRD), Fourier transform infrared (FT-IR) spectroscopy, transmission electron microphotography (TEM), and dynamic light scattering (DLS). Finally, human fibroblasts were cultured in the presence of nanoparticles to evaluate cell proliferation and cytotoxicity.

## Experimental

### Preparation of mercaptoacetic acid-coated CdS quantum dots (CSNPs)

Water-soluble CSNPs were synthesized by following a previously published method [6]. Briefly, carboxyl-stabilized CSNPs were synthesized by arrested precipitation at room temperature in aqueous solution using mercaptoacetic acid as the colloidal stabilizer. Nanocrystals were prepared from a stirred solution of 0.0456 g of CdCl_2 _(5 mM) in 40 ml of pure water. The pH was lowered to 2 with mercaptoacetic acid and then raised to 7 with 1 N NaOH. The mixture was deaerated by N_2 _bubbling for about 30 min, after which 40 ml of freshly prepared 5 mM Na_2_S (0.0480 g of Na_2_S in 40 ml of water) was added to the mixture with rapid stirring. The solution turned yellow shortly after the sulfide addition due to the formation of CSNPs (Scheme 1a in Additional file 1. CSNPs were separated from reaction by-products (sodium salt) via precipitation by the addition of acetone (4 ml of acetone per milliliter of nanocrystal solution). The precipitate was then isolated by centrifugation and dried in a freeze dryer. The prepared powder CSNPs were finally redispersed in water to obtain a clear colloidal solution with excellent stability (zeta potential, -66.65 mV). The free carboxylic acid groups of the prepared CSNPs are suitable for covalent coupling with the primary amino groups of various biomolecules.

### Immobilization of insulin on the surface of CSNPs

Immobilization of insulin on CSNPs was performed in two steps. First, CSNPs were reacted with amino-terminated polyethylene glycol (PEG) to introduce amine groups on their surface. For this, CSNPs (0.2 g) were dissolved in aqueous solution (20 ml) containing 1-ethyl-3-(3-dimethylaminopropyl)carbodiimide (EDC) and stirred for 4 h to activate the carboxylic acid groups on the surface. Then, an excess amount of amine-terminated PEG was added to the solution, which was stirred for 24 h to obtain PEG-grafted CSNPs (PCSNPs) (Scheme 1b in Additional file 1). An excessive amount of PEG was used to suppress the crosslinking reaction on the surface and keep free amine groups at one end of the PEG chain after the reaction [20]. Prepared PCSNPs were isolated via repeated centrifugation and finally dried in a freeze dryer. In the second step, insulin was immobilized on the surface of PCSNPs as follows: insulin was dissolved in phosphate buffer solution (2 mg/ml, pH 7.4) followed by the addition of a small amount of 0.1 N HCl. Then, 2% *w*/*v *water-soluble EDC and NHS were added to the solution, which was incubated at 4°C for 5 h to activate the carboxylic acid groups of the chain. Then, PCSNPs (5 mg/ml) were suspended in phosphate buffer solution (pH 7.4) with vortexing. This PCSNP suspension was mixed with the insulin aqueous solution and stirred gently overnight at room temperature to obtain insulin-immobilized PCSNPs (ICSNPs), as shown in Scheme 1 in Additional file 1. ICSNPs were isolated by repeated centrifugation and stored in phosphate-buffered saline (PBS) at pH 7. All conjugation reactions, unless otherwise noted, were carried out in the dark under a N_2 _ambient environment.

### Surface characterization

Fourier transform infrared (FT-IR) spectra were obtained using a JASCO FT-IR 300E spectrometer (JASCO Inc., Easton, MD, USA) at a resolution of 4 cm^-1^. Dried samples were ground with KBr powder and compressed into pellets for FT-IR examination. The samples were prepared by dropping the diluted nanoparticles on carbon-coated grids, followed by natural drying; then, the samples were observed by a transmission electron microphotograph (Philips CM 200 TEM; applied operation voltage, 120 kV; Philips Inc, Berlin, Germany. The hydrodynamic diameter and size distribution were determined by DLS by means of a standard laboratory-built light scattering spectrometer equipped with a BI 90 particle sizer (Brookhaven Instruments Corp., Holtsville, NY, USA). It had a vertically polarized incident light of 514.5 nm supplied by an argon ion laser (Lexel laser, model 95; Cambridge Lasers Laboratories Inc., Fremont, CA, USA. To investigate the crystal structure of CSNPs and bare CdS, XRD (RA/FR-571, Enraf Nonius, Deift, The Netherlands was used. The result was also compared with Joint Committee on Powder Diffraction Standards (JCPDS) file no. 10-454 to confirm whether or not any impurity phase exists in the CSNPs. The surface chemical composition was analyzed by electron spectroscopy for chemical analysis (ESCA, ESCA LAB VIG microtech, Mt 500/1, etc., East Grinstead, UK) with MgK *α *at 1, 253.6 eV and 150 W of power at the anode. A survey scan spectrum was taken, and the surface elemental compositions relative to the carbon were calculated from the peak heights, taking into account atomic sensitivity. The zeta potential is a very useful way of evaluating the stability of any colloidal system. In this study, the zeta potential was measured with a NicompTM 380 Zeta Potential (ZLS, Tokyo, Japan) employing the electrophoretic light scattering technique and using double-distilled water as a diluent.

### *In vitro *cell behavior

MRC-5 human fibroblast cells (ATCC CCL, 171) were used in this experiment. Cells were routinely cultured at 37°C in a humidified atmosphere of 5% CO_2 _(in air) in a 75-cm^2 ^flask containing 10 ml of Dulbecco's modified eagle medium (DMEM) supplemented with 10% fetal bovine serum (FBS) and 1% penicillin streptomycin G sodium. The medium was changed every 3 days. For subculture, the cells were washed twice with PBS and incubated with trypsin-ethylenediaminetetraacetic acid (EDTA) solution (0.25% trypsin, 1 mM EDTA) for 10 min at 37°C to detach the cells. The cells were washed twice by centrifugation and resuspended in DMEM media containing quantum dot nanoparticles, including CSNPs, PCSNPs, and ICSNPs (particle concentration, 0.2 mg/ml) for reseeding and growing in culture flasks. The cell density was fixed at 1 × 10^5 ^cells/ml. Cell morphologies were observed under a phase contrast microscope (Nikon Eclipse TS100, Tokyo, Japan) at predetermined time intervals.

The proliferation of fibroblasts cultured in the absence and presence of CSNPs, PCSNPs, and ICSNPs was determined by colorimetric immunoassay based on the measurement of 5-bromo-2-deoxyuridine (BrdU), which was incorporated during DNA synthesis [22,23]. BrdU enzyme-linked immunosorbent assay (ELISA; Roche Molecular Biochemicals, Mannheim, Germany) was performed according to the manufacturer's instructions. Briefly, after 48 h of cell culture with CSNPs, PCSNPs, and ICSNPs in 24-well plates, the BrdU-labeling solution was added to each well and allowed to incorporate into the cells for an additional 20 h in a CO_2 _incubator at 37°C. Subsequently, the supernatant in each well was removed by pipetting. The cells were then washed twice with PBS and treated with 0.25% trypsin-EDTA (Gibco, Invitrogen, Tulsa, OK, USA) and harvested by centrifugation at 1, 000 rpm for 15 min. The harvested cells were mixed with a FixDenat solution to fix the cells and denature the DNA, followed by further incubation for 30 min. Subsequently, diluted anti-BrdU peroxidase (dilution ratio = 1:100) was added, and the cells were kept at 20°C for 120 min. After the removal of unbound antibody conjugates, 100 μl of substrate solution was added. The resulting mixture was allowed to stand for 20 min, and the reaction was completed by adding 1 M H_2_SO4 solution. The solution was then transferred to a 96-well plate and measured within 5 min at 450 nm with a reference wavelength of 690 nm using an ELISA plate reader.

### Cytotoxicity

To evaluate the cytotoxicity of Q-dots, the cells were separately cultured in a dish containing CSNPs, PCSNPs and ICSNPs and in a polystyrene culture dish alone. For qualitative observation, Live/Dead fluorescent staining with a LIVE/DEAD Cytotoxicity Kit (Biovision research products, Mountain view, CA 94043 USA) was used. Briefly, fibroblasts (3 × 10^4 ^cell/well) were seeded in a microplate with 1 ml of media containing CSNPs, PCSNPs, and ICSNPs (particle concentration = 0.1 mg/ml) without nanoparticles. After 2 and 4 days of incubation, the media were removed and the cells washed gently with PBS. Then, 0.3 ml of staining solution (prepared by mixing calcein-AM and propidium iodide with staining buffer at a concentration specified by Molecular Probes) was added to each well, and the plate was kept in an incubator for 15 min. Then, calcein/propidium iodide solution was removed, and the cells were washed once again with PBS. Finally, cells were viewed using a fluorescence microscope (FV-300, Olympus Co., Tokyo, Japan) coupled with a digital camera (FV-300, Olympus Co.). Live cells show green fluorescence images and dead cells show red images.

### Statistical analysis

The cell viability experiment was performed in triplicate, and the results are expressed as mean ± standard deviation. Student's *t *test was employed to assess statistical significant difference of the results.

## Results and discussion

### Characterization of surface-modified CdS nanoparticles

**Figure 1:**
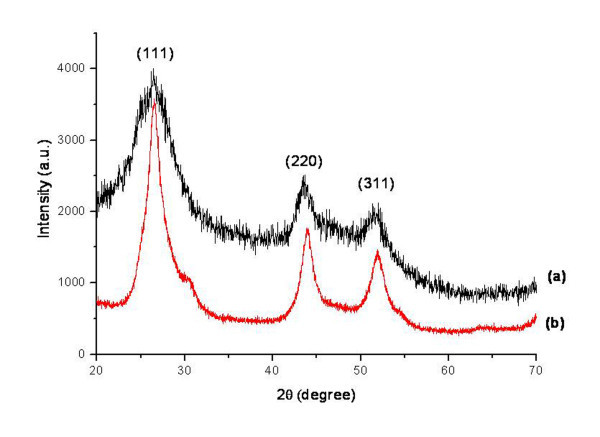
**XRD patterns of (a) mercaptoacetic acid-coated CSNPs and (b) bare CdS**.

**Figure 2:**
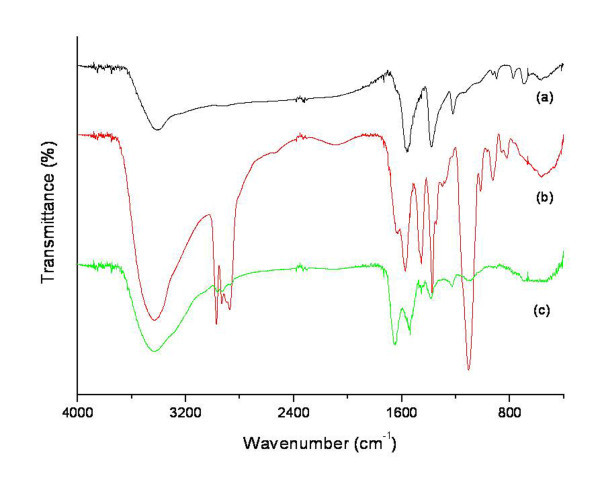
**FT-IR spectra of (a) mercaptoacetic acid-coated CSNPs, (b) PCSNPs, and (c) ICSNPs**.

The X-ray diffraction spectra of CSNPs and bare CdS are shown in Figure 1. It was observed that the number and positions of peaks of CSNPs (Figure 1a) matched well with those of bare CdS (Figure 1b). The spectrum of CSNPs was further compared with the data of JCPDS file no. 10-454 and was in agreement with that of pure cubic-phase CdS, without signals from CdCl_2_, NaOH, or other precursor compounds. The three peaks observed in Figure 1a at 2 *θ *values of 26.439°, 43.862°, and 51.389° were found to correspond to the three crystal planes of (111), (220), and (311), indicating that the CSNPs were in cubic phase [24]. Again, the diffraction peaks of CSNPs were somewhat broad compared to those of bare CdS. This broadness was due to reduced particle size and surface defects [25]. Small-sized CSNPs possess a higher surface defect density due to a high surface-to-volume ratio [26]. Moreover, CSNPs possess higher negative zeta potential (*ξ *= -66.65 mV), indicating excellent stability of the colloidal nanocrystal [27]. The surface modification of CSNPs with insulin was confirmed by FT-IR as displayed in Figure 2. For CSNPs (Figure 2a), two distinctive bands were observed at 1, 559 and 1, 375 cm^-1^, which originated from the asymmetric and symmetric stretching motion of carboxylate ion (-COO^-^) [28]. These findings clearly indicate the formation of a co-ordinate bond between the oxygen atom of mercaptoacetic acid and Cd^+2^. No free carboxylic acid band at 1, 730 to 1700 cm^-1 ^due to C=O stretching is observed in capped nanoparticles [29]. The introduction of PEG onto the surface of CSNPs was confirmed by the characteristic peak at 1, 575 cm^-1^, which can be attributed to a -CH_2 _bending vibration (Figure 2b). Besides, a peak at 1, 106 cm^-1 ^indicated an ether bond (-C-O-) of PEG. Some other peaks were observed at positions of 2, 972, 1, 455, and 1, 375 cm^-1^, which originated from the PEG chain. This implies that the basic structure of PEG did not change, except for the conversion of a terminal group [17]. After reaction of PEG-immobilized CSNPs (PCSNPs) with insulin, two new peaks at positions around 1, 648 and 1, 540 cm^-1 ^were observed based on -CO-NH- (amide I) and -CO-NH- (amide II) bands, respectively [20] (Figure 2c). These results indicate that insulin was successfully immobilized onto the surface of PCSNPs.

**Figure 3:**
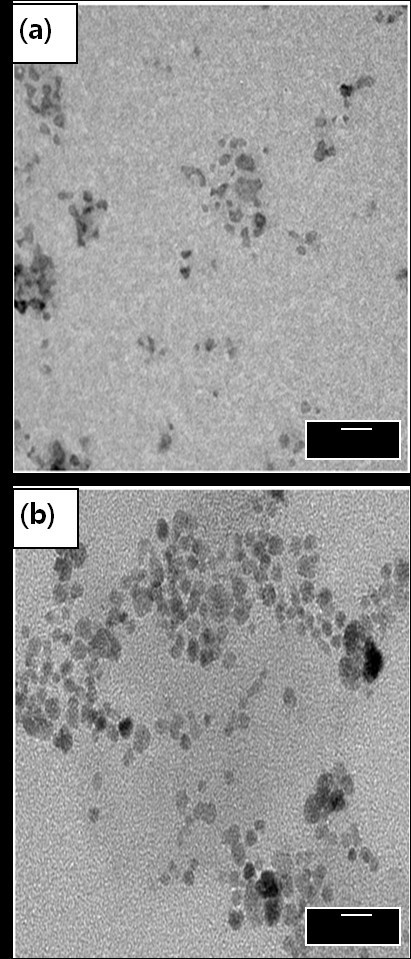
**TEM images of (a) mercaptoacetic acid-coated CSNPs and (b) ICSNPs**.

**Figure 4:**
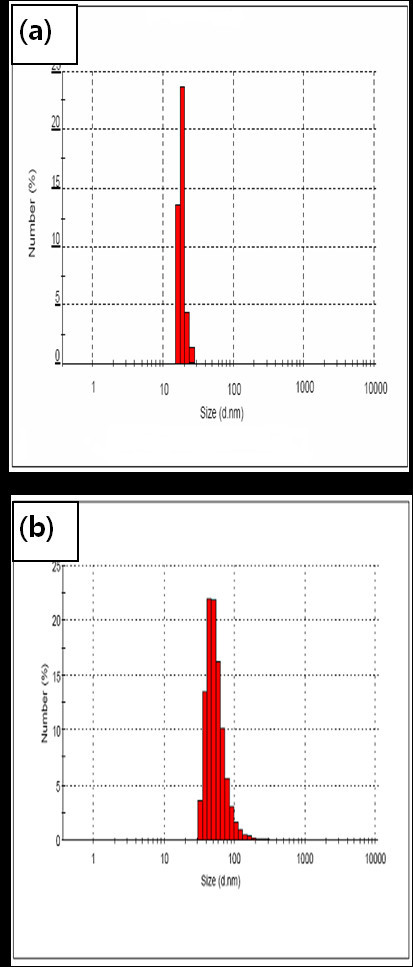
**Particle size distributions of (a) mercaptoacetic acid-coated CSNPs and (b) ICSNPs**. As measured by dynamic light scattering.

Immobilization of insulin onto the surface of CSNPs was further confirmed by ESCA. The chemical compositions of CSNPs, PCSNPs, and ICSNPs, as calculated from the ESCA survey scan spectra, are shown in Table 1. In the case of PCSNPs, the oxygen content (22.09%) and carbon content (62.09%) were higher in comparison to those of CSNPs (oxygen content, 17.35% and carbon content, 30.04%). Furthermore, one new element such as nitrogen (1.59%) was observed on the surface of PCSNPs, indicating the successful immobilization of PEG onto the surface of CSNPs. In the case of ICSNPs, nitrogen content increased from 1.59% to 2.72% and oxygen content increased from 22.09% to 35.81%, indicating the successful immobilization of insulin onto the surface of PCSNPs. TEM images of CSNPs and ICSNPs are shown in Figure 3. It was observed that CSNPs had spherical morphologies with an average diameter of *ca*. 4.5 nm. Due to the small dimensions and high surface energy of the particles, it was easy for them to aggregate as seen in Figure 3a. On the other hand, immobilization of insulin conferred a spherical morphology, thereby reducing the aggregation of particles. The average diameters of the ICSNPs were 13 nm as shown in Figure 3b. Larger diameters and lower aggregation of particles may have resulted from the immobilization of insulin and PEG onto the surface of CSNPs. Figure 4 shows the typical size and size distribution of synthesized CSNPs (Figure 4a) and ICSNPs (Figure 4b) as measured by DLS. The average size of CSNPs as determined by DLS was *ca*. 21 nm. On the other hand, the average sizes of ICSNPs were about 38 nm. The size of the particles as determined by DLS was considerably larger than that determined by TEM, most likely because the DLS technique gives the mean hydrodynamic diameter of the core of CSNPs surrounded by organic and solvated layers, which is influenced by the viscosity and concentration of the solution. On the other hand, TEM gives the diameter of the core alone [30].

**Table 1:** Atomic percent of CSNPs, PCSNPs, and ICSNPs calculated from ESCA survey scan spectra

Sample	Atomic percent (%)				
	C	O	N	S	Cd
CSNP	30.04	17.35	-	16.26	36.35
PCSNP	62.09	22.09	1.59	6.71	7.52
ICSNP	56.79	35.81	2.72	1.33	3.35

### Cell proliferation

**Figure 5:**
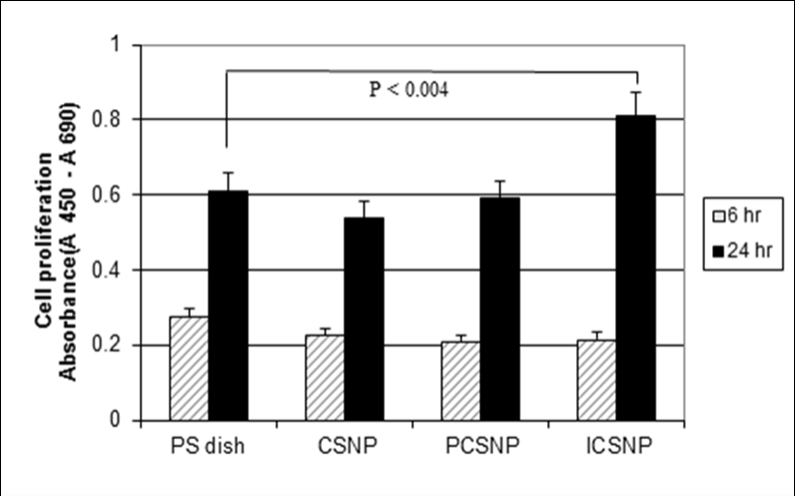
**Proliferation of human fibroblasts after 6 and 24 h of incubation**. In a dish containing CSNPs, PCSNPs, and ICSNPs and in a polystyrene culture dish, as measured by BrdU assay.

**Figure 6:**
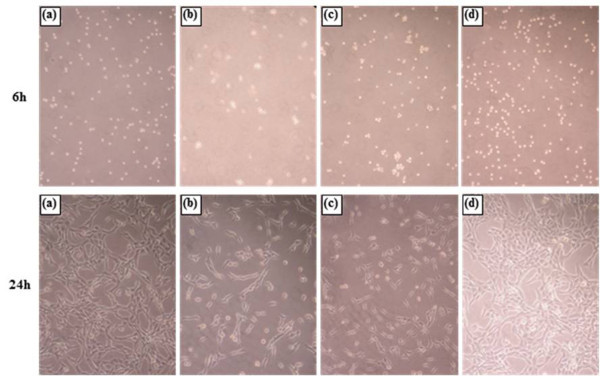
**Optical microscopic images of human fibroblasts cultured for 6 and 24 h**. In a (**a**) polystyrene culture dish and in the presence of (**b**) CSNPs, (**c**) PCSNPs, and (**d**) ICSNPs. Original magnification is ×200.

The proliferation of fibroblasts was evaluated by two different methods, BrdU assay and morphological observation. Figure 5 shows the pattern of fibroblast proliferation as measured by BrdU assay after 6 and 24 h of culture in media containing CSNPs, PCSNPs, and ICSNPs. A significant difference in the acceleration of cell growth was not observed after 6 h of culture with CSNPs (Figure 6b), PCSNPs (Figure 6c), ICSNPs (Figure 6d), or without nanoparticles (Figure 6a). This could be attributed to the non-interference of particles during the short incubation period. In this case, fibroblasts adhesion occurs due to the influence of FBS-containing media only. However, after 24 h of culture, cell proliferation in media containing ICSNPs was significantly accelerated compared to that in media only. However, cell proliferation in media containing PCSNPs or CSNPs was not significantly different from that in media only (*p *< 0.004). Fibroblast proliferation was suppressed in media containing CSNPs. This low cell proliferation was probably due to the negative charge of the surface carboxyl groups on CSNPs [19]. Cell proliferation in media containing PEG-immobilized CSNPs (PCSNPs) was almost the same as that in the control culture dish. This was because the biocompatibility of PEG has already been proven [17]. On the other hand, fibroblast proliferation in media containing ICSNPs was the highest, probably because immobilized insulin molecules sufficiently and continuously stimulate receptors expressed on the plasma membrane surface as well as downstream signal transduction proteins without internalization of ligand-receptor complexes [18]. These results suggest that the binding of immobilized insulin with insulin receptors is essential for the acceleration of the cell proliferation. Similar studies have been reported elsewhere [31,32]. Kim et al. [20] prepared insulin-immobilized polyurethanes and evaluated their interaction with human fibroblasts. As a result, cells were more rapidly proliferated onto insulin-immobilized polyurethanes compared to that on both polyurethane (PU) control and PEO-grafted PU when cultured in the presence of serum. Cell proliferation in the presence of CSNPs, PCSNPs, and ICSNPs and in the absence of nanoparticles was further visualized using an optical microscope. As a result, cell proliferation in the presence of ICSNPs was found to be higher than that in the presence of CSNPs and PCSNPs, as shown in Figure 6.

### Cellular cytotoxicity

**Figure 7:**
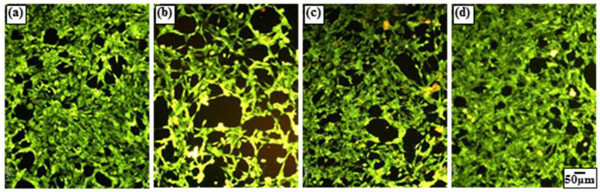
**Fluorescence microscopic images of Live/Dead dye-stained fibroblasts cultured for 2 days**. In a (**a**) polystyrene culture dish and in the presence of (**b**) CSNPs, (**c**) PCSNPs, and (**d**) ICSNPs. Live and dead cells were stained and visualized in green and red, respectively, under a fluorescence microscope.

The status of the ''Live/Dead'' dye-stained fibroblasts cultured in the presence of CSNPs, PCSNPs, and ICSNPs and in the absence of nanoparticles for 2 days is shown in Figure 7. Using this qualitative method, live and dead cells were stained green and red, respectively, under a fluorescence microscope. The color of the cells cultured on a PS dish and with ICSNPs was green, indicating good viability. On the other hand, when cultured with CSNPs and PCSNPs, the green color was partially mixed with red, showing that some parts of the cells were dead. Possible explanations are: (1) toxic cadmium ion (Cd^+2^) release from CSNPs due to surface oxidation causes cell death [4,33]; (2) reactive oxygen species (ROS) react with cellular biomolecules, resulting in damage, degradation, and finally loss of function [34,35]; (3) nanoparticles are taken up by the cells as a result of endocytosis, which results in disruption of the cell membrane [36]; or (4) weak cell adhesive interactions with CSNPs promote apoptosis (programmed cell death) [36]. Since mercaptoacetic acid is the least solubilizing ligand, and it alone could not protect CSNPs from surface oxidation and diffusion of Cd^+2 ^ions from CSNPs over a longer period, it is difficult to make CSNPs biological inert. Therefore, most of the cells died in the presence of CSNPs [4,33,34]. Again, the cell viability of PCSNPs was moderately low due to the surface immobilization conferred by biocompatible PEG, which reduced the release of cadmium ion (Cd^+2^) and formation of ROS. On the other hand, ICSNPs revealed no cytotoxic effects on cells for up to 2 days. This increased cell viability can be explained by a nutrient effect [36,37]. Besides, the low toxicity of nanoparticles immobilized with insulin may be attributed to the fact that these ligands act as cellular markers that target surface receptors expressed on the cell surface without being internalized. Receptors are highly regulated cell surface proteins that mediate specific interactions between cells and their extracellular milieu, and they are generally localized to the plasma membrane [36]. Based on this explanation, it could be said that the immobilization of biomolecules onto the surface of Q-dots can suppress their toxicity. In this study, PEG and insulin in combination reduced the cytotoxicity of cells.

## Conclusions

Insulin was immobilized onto the surface of mercaptoacetic acid-coated cadmium sulfide nanoparticles (CSNPs), and confirmation of insulin immobilization was carried out by FT-IR and ESCA. Size distribution of insulin-immobilized CSNPs (ICSNPs) having an average diameter of 13 nm as determined by TEM was narrow. The proliferation of fibroblasts was significantly increased by the presence of ICSNPs. ICSNPs also demonstrated lower cytotoxicity than CSNPs and PCSNPs. The ICSNPs used in this study will have potentials to be used in bio-imaging applications.

## Competing interests

The authors declare that they have no competing interests.

## Authors' contributions

KMK carried out the preparation and immobilization research work. ZCX and MJC participated in the data processing. YC, HG, IKK participated in the design of the study and performed the statistical analysis. All authors read and approved the final manuscript.

## Supplementary Material

Additional file 1 **Scheme 1**. Schematic diagram showing the preparation of (a) mercaptoacetic acid-coated CSNPS, (b) PCSNPs, (c) ICSNPS and (d) bare CdS. Click here for file
