# Supplementary figures and images for: Reduced cytotoxicity of insulin-immobilized CdS quantum dots using PEG as a spacer
Source: Nanoscale Res Lett. 2011 Sep 23;6(1):528. doi: 10.1186/1556-276X-6-528 (PMC3228607; doi:10.1186/1556-276X-6-528)

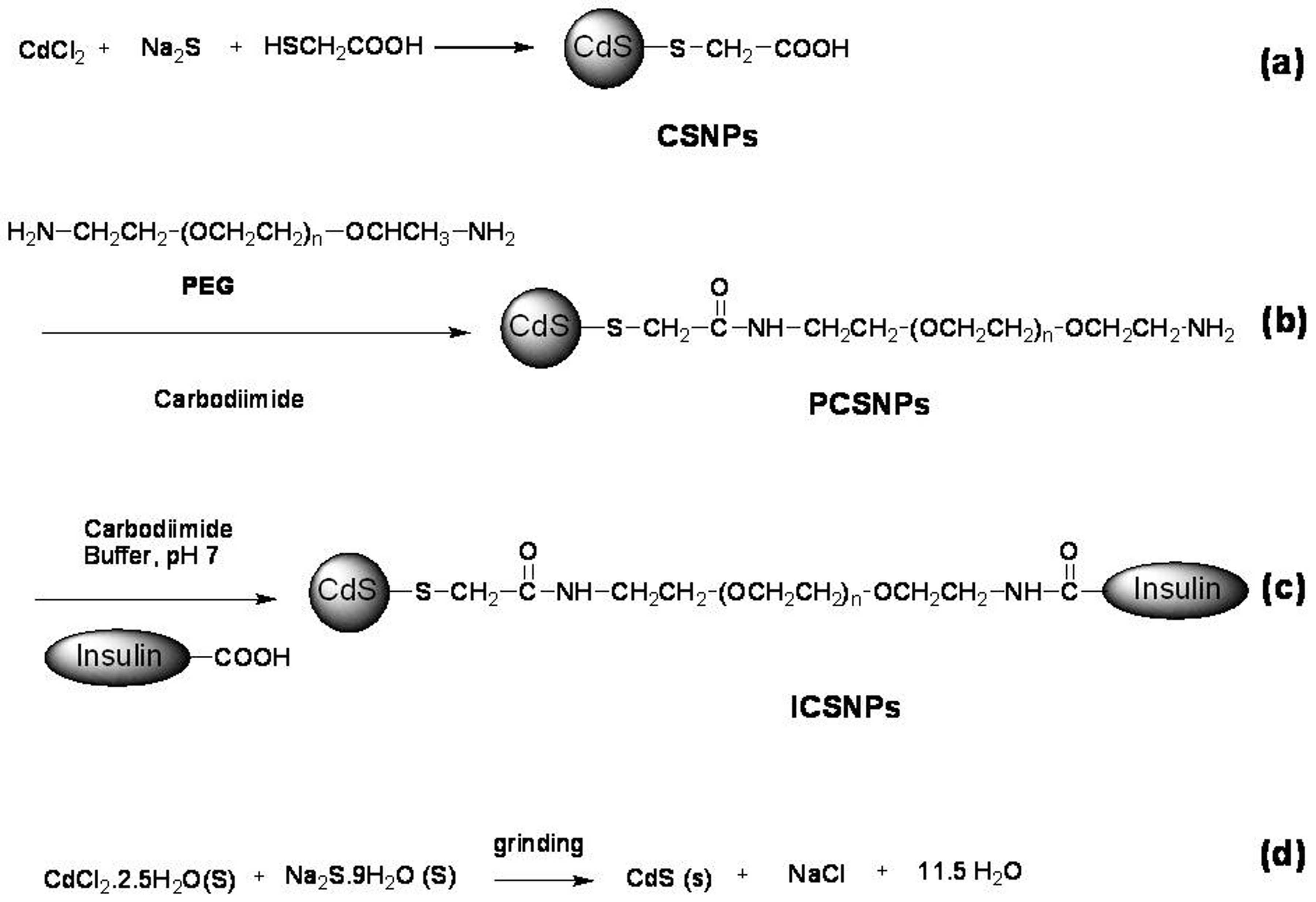

Supplement: Additional file 1 — Scheme 1. Schematic diagram showing the preparation of (a) mercaptoacetic acid-coated CSNPS, (b) PCSNPs, (c) ICSNPS and (d) bare CdS. [file 1556-276X-6-528-S1.jpeg]
